# Evidence and mapping of extinction debts for global forest-dwelling reptiles, amphibians and mammals

**DOI:** 10.1038/srep44305

**Published:** 2017-03-16

**Authors:** Youhua Chen, Shushi Peng

**Affiliations:** 1 Department of Renewable Resources, University of Alberta, Edmonton, T6G 2H1, Canada; 2 Sino-French Institute for Earth System Science, College of Urban and Environmental Sciences, Peking University, Beijing, 100871, China

## Abstract

Evidence of extinction debts for the global distributions of forest-dwelling reptiles, mammals and amphibians was tested and the debt magnitude was estimated and mapped. By using different correlation tests and variable importance analysis, the results showed that spatial richness patterns for the three forest-dwelling terrestrial vertebrate groups had significant and stronger correlations with past forest cover area and other variables in the 1500 s, implying the evidence for extinction debts. Moreover, it was likely that the extinction debts have been partially paid, given that their global richness patterns were also significantly correlated with contemporary forest variables in the 2000 s (but the absolute magnitudes of the correlation coefficients were usually smaller than those calculated for historical forest variables). By utilizing species-area relationships, spatial extinction-debt magnitudes for the three vertebrate groups at the global scale were estimated and the hotspots of extinction debts were identified. These high-debt hotspots were generally situated in areas that did not spatially overlap with hotspots of species richness or high extinction-risk areas based on IUCN threatened status to a large extent. This spatial mismatch pattern suggested that necessary conservation efforts should be directed toward high-debt areas that are still overlooked.

Extinction debt describes a delayed process of species extinction after habitat destruction^1^,^2^,^3^,^4^. The existence of an extinction debt can be supported when the correlation between contemporary spatial species diversity patterns and the historical environmental conditions is much higher than the correlation between contemporary spatial species diversity patterns and the contemporary environmental conditions^5^. Many empirical studies have contributed to the evidence and knowledge of extinction debts in ecological communities at the local and landscape levels^5^,^6^,^7^,^8^, but few studies have been conducted to test and predict extinction debts at large spatial scales^4^.

The evidence for extinction debt can be measured in multiple ways^9^. The most important one is comparing the correlations between contemporary species richness versus past and present habitat variables (e.g., area size). If there is a significant relationship between past habitat size and species richness but a non-significant relationship between current habitat size and richness, it is believed that the evidence for extinction debts exists. The absolute magnitude of correlation coefficients is also important in identifying debts. Evidence for extinction debt is also supported if the correlation coefficient between species richness and past habitat size has a large absolute value. Species-area relationships (SARs) and regression analyses have also been widely used to evaluate the existence of extinction debts^9^,^10^, but the basic idea is very similar to correlation tests (i.e., also checking the significance and magnitude of the fitted coefficients).

Land use change has been identified as a top factor in addition to climate change^11^,^12^ in the evaluation of biodiversity responses and extinction risks for species in a changing environment^13^,^14^. Because of accelerating anthropogenic interferences, land use change more or less results in the fragmentation, destruction and pollution of the habitats, increasing the likelihood of species extinction^15^,^16^,^17^. Using land use history to estimate extinction debts has been a common practice in the conservation literature^1^,^4^,^9^.

One top variable in assessing the impacts of land use change on species extinction is forest change. In the present study, by using a novel data set on the forest cover dynamics at a global scale dated back to the 1500 s, we are interested in finding the evidence of extinction debts for global forest-dwelling terrestrial animals (including amphibians, mammals and reptiles), as well as in estimating their magnitude and mapping their spatial distributions. The ultimate goal of our study is to identify potential conservation gaps derived from the spatial extinction-debt patterns.

To test the signals of extinction debts for global vertebrate assemblages, different correlation tests were applied. If there are indeed signals of extinction debts, the corresponding spatial extinction debt patterns estimated at the global scale on the basis of forest cover-derived SAR models would be legitimate. The following questions will be addressed accordingly: (1) Is there clear evidence of extinction debts for global forest-dwelling amphibians, reptiles and mammals? (2) What is the relative importance of different forest variables in explaining contemporary species richness patterns? (3) Where are the hotspots with a high magnitude of extinction debts for each vertebrate group? (4) Are these extinction-debt hotspots spatially congruent with hotspots of species richness or extinction risk based on IUCN (http://www.iucnredlist.org/) threatened status? (5) Where are the potential conservation gap areas?

## Results

**Figure 1:**
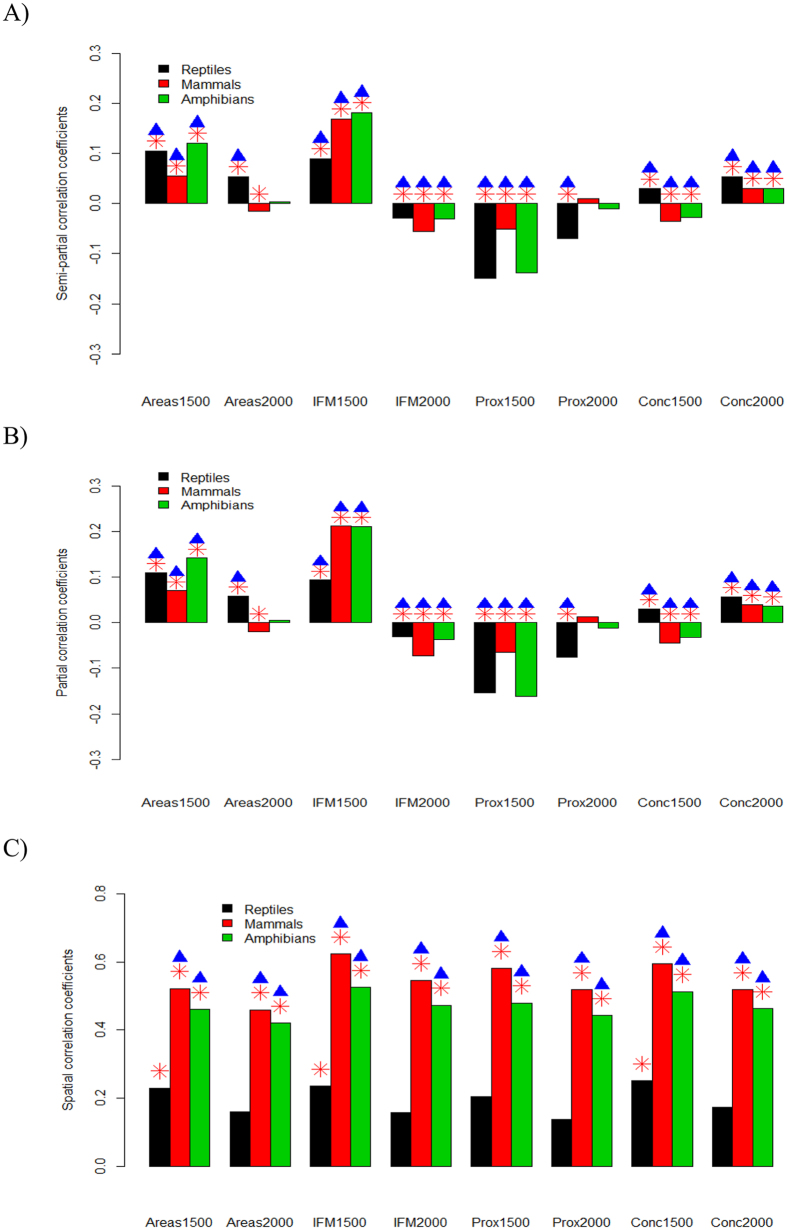
Semi-part (**A**), partial (**B**) and spatial (**C**) correlations between each past or current forest cover-relevant variable and current species richness patterns while controlling the effects of other forest cover-relevant variables. Y-axis always denotes the correlation values. Stars on top of the bars indicate significant correlations (*P < 0.05*). Codes: past forest cover size (Areas1500); current forest cover size (Areas2000); past forest connectivity (IFM1500); current forest connectivity (IFM2000); past forest proximity (Prox1500); current forest proximity (Prox2000); past forest concentration (Conc1500); current forest concentration (Conc2000). These maps are created using R software (version 3.2; https://www.r-project.org/)^36^.

Both semi-part and partial correlations had very similar results (Fig. 1A,B). Specifically, amphibian and mammal richness were always significantly correlated with the past but not the current forest area size, particularly when the *p*-values were adjusted (Fig. 1A,B). Moreover, they were significantly correlated with the past but not the present forest proximity (Fig. 1A,B). In the spatial correlation test, when the Bonferroni correction was not applied, reptile richness was significantly associated with the past but not the current forest area size, connectivity and concentration (Fig. 1C). Negative correlations occurred when the historical and current forest proximity, current connectivity and historical concentration were involved in the semi-part and partial correlation tests, but not the spatial correlation test (Fig. 1).

**Figure 2:**
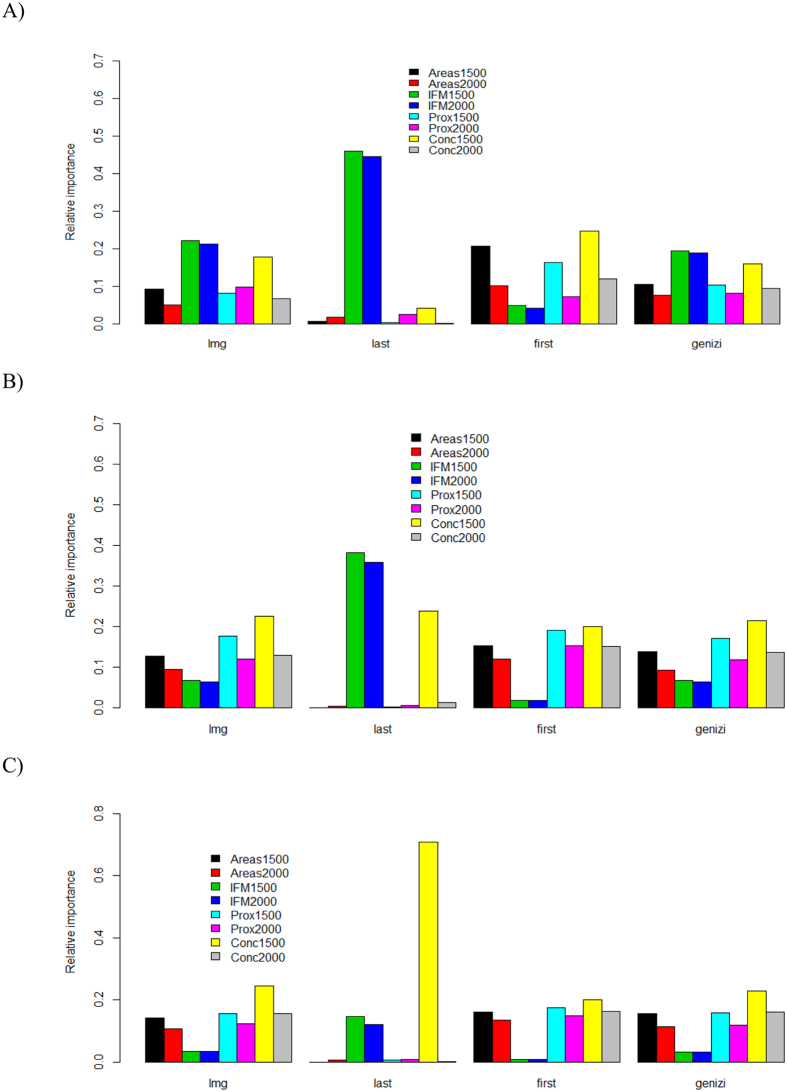
Variable importance of each forest cover-related predictors on explaining global richness patterns of reptiles (**A**), mammals (**B**) and amphibians (**C**) respectively. Codes: lmg: the R^2^ contribution averaged over orderings among regressors; last: the contribution of each variable when included last in the model; first: the contribution of each variable when included first in the model (first); genizi: the R^2^ decomposition according to a previous study^38^. These maps are created using R software (version 3.2; https://www.r-project.org/)^36^.

For all the three kinds of correlation tests, most of the past forest variables, except for the concentration index, were strong and significant predictors of the contemporary global vertebrate richness patterns, regardless of whether the *p*-values were adjusted or not (Fig. 1). Of course, the current forest variables were also significantly correlated with species richness in the tests (Fig. 1). For example, the current forest connectivity and concentration indices were significantly related to the species richness of all the three vertebrate groups (Fig. 1A,B). However, when the Bonferroni correction was applied, the number of significant correlations between the current forest variables and species richness was reduced (Fig. 1). More importantly, the absolute magnitudes of the correlation coefficients (or effective size; whether they were statistically significant or not) for the current forest variables were usually smaller than those for the past forest variables in the 1500 s, irrespective of the correlation test used (Fig. 1). Such an observation was further confirmed in the variable importance analyses: in most cases, past forest cover variables had higher relative importance than current forest variables across the three taxonomic groups (Fig. 2A–C).

The randomization test on the statistical significance of the observed correlation values showed very similar results (Supplementary Table S1). Vertebrate species richness had non-significant associations with present forest variables in the semi-part and partial correlation tests, specifically between mammal and amphibian richness versus current forest area size and proximity (Supplementary Table S1). By contrast, all observed spatial correlation values were found to be significantly different from those that were randomly generated (Supplementary Table S1).

With respect to the relative importance of the past and current forest cover-relevant variables, as evidenced by the different ranking metrics (lmg, last, first, and genizi) in the variable importance analyses (Fig. 2), the past concentration index (CONC1500), usually followed by past and current forest connectivity (IFM1500 and IFM2000), were the top three variables explaining global forest-dwelling vertebrate richness.

**Figure 3:**
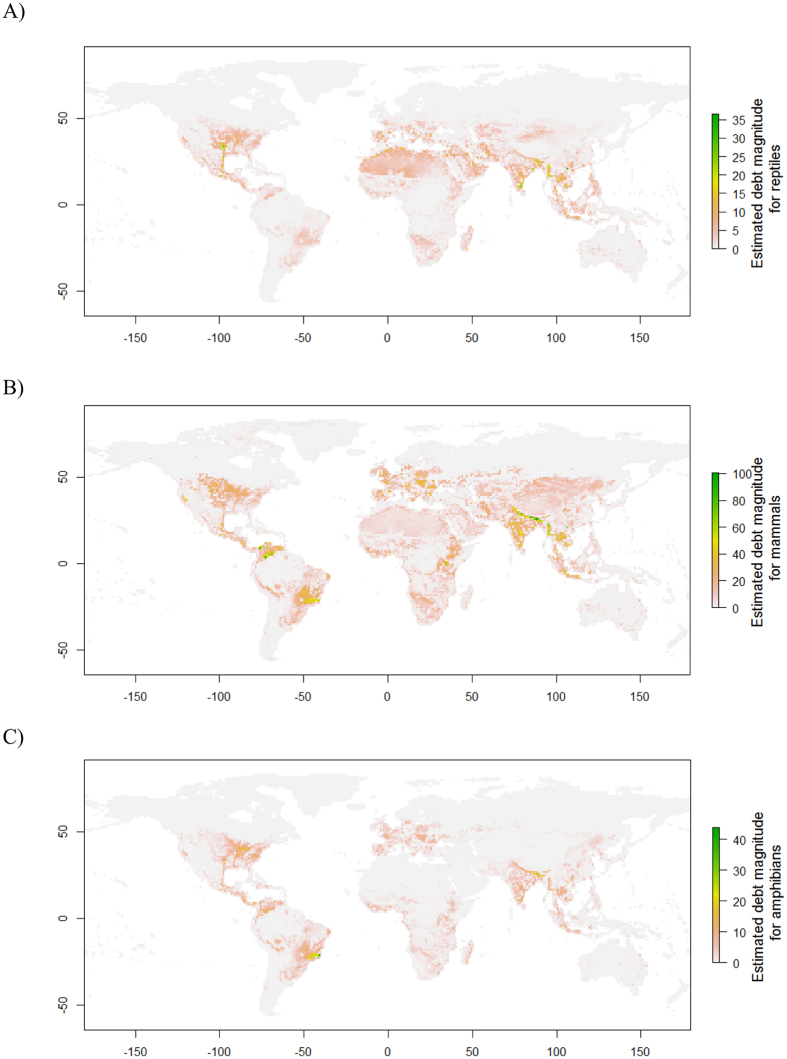
Estimated spatial extinction debt magnitude for global forest-dwelling reptiles (**A**), mammals (**B**) and amphibians (**C**). These maps are created using R package “raster” (version 3.2; https://www.r-project.org/)^36^.

Using SARs, the estimated magnitude of the extinction debts based on forest cover change for the three taxonomic groups is shown in Fig. 3. Applying different *z* values (either the predefined values 0.1 and 0.15 or those directly fitted from the data) to the SARs only changed the debt magnitude but did not alter the spatial arrangements of the high-magnitude areas, regardless of the taxonomic group studied (Fig. 3 versus Supplementary Fig. S2). Thus, it was reasonable to only utilize the debt-magnitude maps (Fig. 3) generated by the SAR models with *z* = 0.25 for subsequent analyses and comparisons.

**Figure 4:**
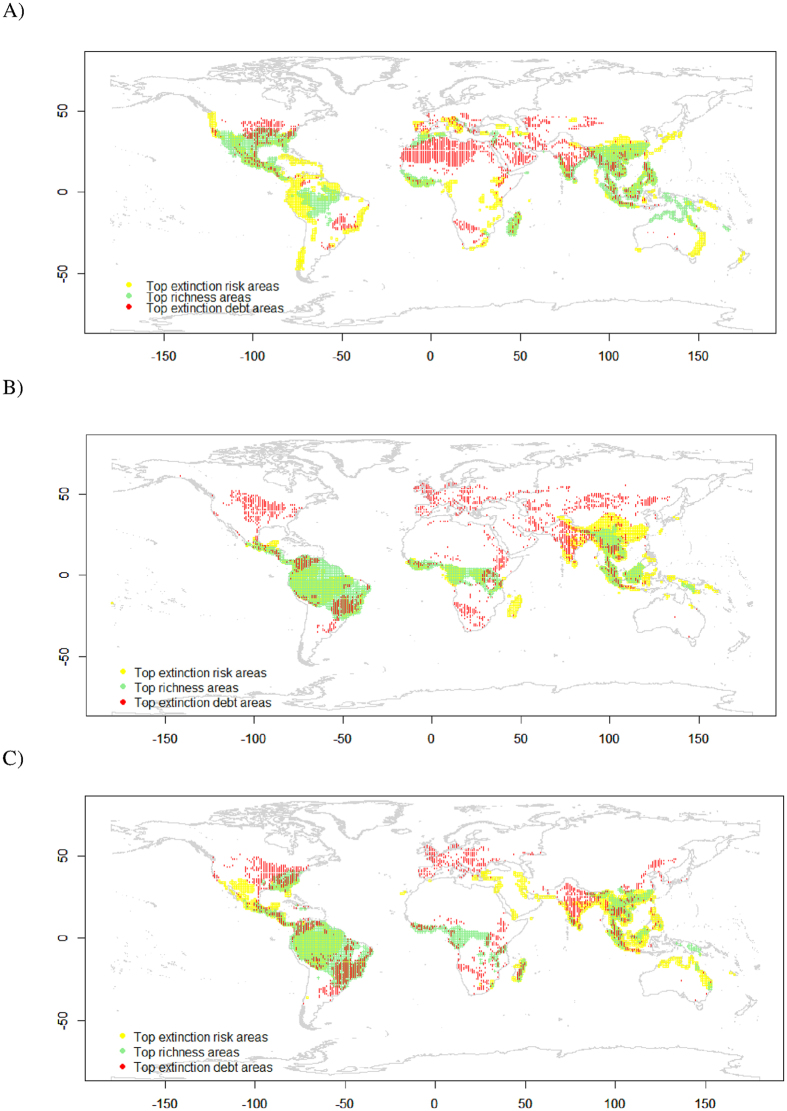
Spatial mismatch between top 10% areas with the highest species richness (in green color), the highest extinction risk (in yellow color), or the highest extinction debt magnitude (in red color) for global forest-dwelling reptiles (**A**), mammals (**B**) and amphibians (**C**). These maps are created using R package “raster” (version 3.2; https://www.r-project.org/)^36^.

To a great extent, the top 10% highest extinction-debt areas did not spatially overlap with species richness hotspots and IUCN-based high extinction-risk areas (Fig. 4). In particular, some top-ranked areas with a very high extinction-debt magnitude, specifically those found in southern Europe, southern Africa, India and the eastern USA, were not covered by any of the top 10% richness hotspots or IUCN extinction-risk hotspots, regardless of the taxonomic group studied (Fig. 4A–C).

For reptiles, areas with a high magnitude of extinction debt were situated in southeastern and western Asia, India, the eastern USA, and the Mediterranean and its adjacent regions (Figs 3A and 4A). Comparatively, both richness hotspots and high extinction-risk areas for reptiles were usually located in Central and South America, Madagascar, and a wide area from southern China to southeastern Asia and northern Australia (Fig. 4A, Supplementary Figs S1A and S3A).

For mammals, areas with high extinction debts were found in western and northern Europe, India, northern and southeastern South America, and the eastern USA (Fig. 3B). Other top 10% high-debt areas can be found in northern China and Mongolia, as well (Fig. 4B). In contrast, the hotspots of species richness were situated in the range from Mexico to the Amazon rainforests and eastern South America, in central Africa and from southeastern Asia to the Malay Archipelago (Fig. 4B or Supplementary Fig. S1B). Finally, hotspots with very high extinction risk based on IUCN threatened status were concentrated in southeastern Asia and Madagascar (Supplementary Fig. S3B). Other top 10% areas can be found in southern China and India (Fig. 4B).

For amphibians, areas with a high extinction-debt magnitude were located in the broad range from the eastern USA to eastern South America (except the Amazonian rainforest), western and eastern Europe, India and from northeastern China to southeastern Asia (Fig. 3C). By comparison, richness hotspots were found in the ranges from the Amazon rainforests to eastern South America, from southern China to southeastern Asia, and in central Africa and Madagascar (Fig. 4C or Supplementary Fig. S1C). Finally, very high extinction-risk areas were in the range from the Caribbean to the northern part of the Andes Mountains (Supplementary Fig. S3C). Other top extinction-risk areas can also be found from southeastern Asia to eastern coastal areas of Australia (Fig. 4C).

## Dsicussion

The present study found that there was evidence of extinction debts for global forest-dwelling terrestrial amphibians, mammals and reptiles. Moreover, the evidence was weak, and it was possible that the debts across all three taxonomic groups might be partially paid, in that species richness was also occasionally significantly correlated with current-time forest variables (Figs 1 and 2). High extinction-debt areas were identified based on SAR models (Figs 3 and 4), and these areas should be given higher conservation priority because many of them have not been spatially represented (or covered) by any well-informed conservation-important areas (i.e., current richness hotspots and top IUCN extinction-risk areas) (Fig. 4). These overlooked areas with a high debt magnitude were consistently found in southern Europe, southern Africa, India and the eastern USA for all three taxonomic groups (Fig. 4).

As mentioned above, negative correlations between species richness and some forest variables (i.e., forest proximity) were found in the semi-part and partial correlations (Fig. 1). This result is largely due to the statistical properties of these correlation tests, the aims of which are to control the influence of other variables. Because different variables are often correlated, the removal of the influence of other variables on the focal variables is equivalent to the use of the residuals of the focal variables in ordinary correlation analyses. Such an operation could result in either negative or positive correlation signs. When the influence of other forest variables was not controlled for (e.g., in spatial correlation or ordinary Pearson product-moment tests), the correlations between forest variables and species richness became positive (Fig. 1C), following the conventional expectations.

It is worth mentioning that the empirical *z* value for global reptiles has not been previously reported^18^. For the first time, we reported here that the *z* value for global reptiles was actually very small (=0.035, see the legend of Supplementary Fig. S2C) and quite different from the fitted *z* values for the other two vertebrate groups (*z* = 0.22 for mammals and 0.31 for amphibians, see the legends of Supplementary Fig S2F and S2I). Interestingly, although forest-dwelling reptiles presented a weak relationship with forest cover area size (indicated by the small fitted *z* value in the SAR above), they presented the strongest signal of extinction debts among the three taxonomic groups in the spatial correlation test whenever the *p*-value was not adjusted (Fig. 1C).

SAR models are a basic and powerful tool for estimating extinction debts, in which the size of the sampling areas should be clearly defined. In our work, we estimated extinction debts for forest-dwelling vertebrates based on SAR models constructed using forest cover as the sampling areas (Fig. 3). Habitat area size is the most important variable in the evaluation of extinction debts, as reported in many studies at the local and landscape levels^1^,^4^,^9^,^15^. Our study takes a step forward and might be the first to apply forest cover area in assessing extinction-debt patterns at the global scale.

There is another common way to estimate extinction debts that has not yet been mentioned above: species distribution models (SDMs)^19^,^20^,^21^. However, there are uncertainties brought about by SDMs, ranging from the modeling algorithms to the climate data used^15^,^22^,^23^. Additionally, the computation is very intensive and time-consuming. By contrast, SAR models are very simple and fast to calculate, as long as the sampling area data are ready (i.e., forest cover-based land use data in our study). Moreover, the estimation uncertainty of extinction debts brought about by species-specific range dynamic modeling using SDMs can be avoided.

A practical but challenging problem that remains unsolved in terms of estimating extinction debts is that we are unable to quantify the relaxation time of the debts. Knowing the length of the time window for fulfilling extinction debts has important conservation implications, as species can be conserved only before they are completely extirpated. One way to estimate the extinction time is to construct dynamic SARs^4^. However, there are uncertainties brought about by these dynamic models, as the relaxation parameter in the models^4^ has been little reported in previous empirical studies. Another way to estimate the relaxation time is to use metapopulation models^24^,^25^,^26^,^27^, but a similar problem occurs: the extinction and colonization parameters have to be given or estimated.

Our study may be the first report identifying the evidence and mapping the spatial distribution of extinction debts at the global scale. However, we also recognize that there are limitations that should be discussed and may note the pathways for further investigations. First, similar to the projected climate data, there were uncertainties in the estimation of historical forest cover back to the 1500 s. As the extinction-debt magnitude in our study strongly relied on the quality of forest cover data by using SAR models, it is necessary to evaluate the potential influences of different historical forest change scenarios on the estimation of the debts. Second, historical species richness may be quite different from the current-time species richness. Over the past 500 years, species’ distributional ranges may have changed frequently, and the estimation of extinction debts using current species richness values may be insufficient. A possible solution for this is to project species’ historical ranges using SDMs and utilize the derived historical and current species richness for estimating debts. Finally, the IUCN spatial data are inexact, and the distributional ranges of some species are inaccurate or even missing. To overcome this, it is necessary for global ecologists to work together and cumulatively contribute knowledge on the occurrence of species worldwide, in particular from field surveys and for threatened species, to continue to improve the quality of data on species’ distributions at broad scales.

In conclusion, the present study showed that global forest-dwelling amphibians, reptiles and mammals may be subjected to weak or partially paid extinction debts. Based on SAR models, it was found that the areas with a high debt magnitude were not overlapped with richness hotspots or top IUCN extinction-risk areas to a great extent (Fig. 4), implying the necessity of allocating more conservation efforts to these overlooked areas.

## Methods

### Global forest-dwelling amphibian, reptile and mammal distributions

The global spatial distributional data for amphibians, reptiles and mammals were compiled from an online database (http://www.iucnredlist.org/technical-documents/spatial-data). In our study, based on the IUCN habitat information for each species, we only selected terrestrial forest-dwelling species in each taxonomic group for the subsequent analyses. Correspondingly, we excluded species inhabiting marine, freshwater or other non-forest habitats. We then overlapped the distributional range of each species over a grid system with a spatial resolution of 1° × 1° geographic degrees that covered the global land surface (but excluding Antarctica) to record its presence/absence information in each grid cell. The cumulative presence-absence information for all the species was used to generate spatial richness patterns (Fig. S1) and the subsequent analyses.

### Global forest cover

Past and current global forest cover data for the 1500 s and 2000 s with a spatial resolution of 1° × 1° were generated from the Land Use Harmonization (LUH; version 3.1) database (http://luh.umd.edu/data.php)^28^ and the European Space Agency CCI land cover map available at the current time (for the year 2005; http://www.esa-landcover-cci.org/) with a backward algorithm described in Peng *et al*. (*submitted*). Specifically, starting from the CCI land cover map, we estimated the annual changes in different land use types between two consecutive years based on the LUH data using a backward “proportional allocation rule”^29^. That is, the annual historical changes in urban, cropland and pasture areas in each grid cell were calculated as percentages in a backward and recursive way back to 1500 A.D. using the ratios of existing natural grassland to forest for different land use types^30^. The historical gain or loss of forest cover thus was proportionally related to the overall changes in urban, cropland and pasture areas^29^.

We utilized historical and current forest cover data from the 1500 s and 2000 s, respectively, when evaluating the possible existence of extinction debts for global vertebrate species. To reduce uncertainty, the forest cover maps from the 1500 s and 2000 s were taken as the mean of dynamic forest cover data spanning a 50-year interval from 1500 A.D. to 1550 A.D. and 1950 A.D. to 2000 A.D., respectively. The fraction of the forest cover in each grid cell was used to compute the forest area for each cell by multiplying the total areal size of the cell. Forest area sizes at the past (1500 s) and present (2000 s) times for cell *i* are designated *A*_1500_(*i*) and *A*_2000_(*i*), respectively. They are also coded as Areas1500 and Areas2000, respectively, in the figures and tables.

### Forest connectivity, proximity and concentration indices

In addition to the forest area size, we also computed some important variables that are thought to be closely related to the occurrence of species based on metapopulation theory and spatial ecology, which were forest connectivity, proximity and concentration indices^31^.

Forest connectivity across the grid cells can influence species distributions^6^. This index quantifies the colonization potential of species in the forest patches^32^. Intensified forest fragmentation (and thus low connectivity of forest patches) would result in a high isolation level of species’ populations and correspondingly a high extinction risk for the species. The forest patch connectivity (IFM) for a specific cell *i*^6^ using past or current forest cover is calculated as^32^,^33^


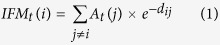


where *t* = the 1500 s or 2000 s and *d*_*ij*_ is the geographic distance between the two cells *i* and *j*.

The forest proximity index measures the distance of the surrounding cells to a focal cell by integrating the forest area size in each cell^31^. In our study, the forest proximity for a specific grid cell *i* in the 1500 s and 2000 s is given by


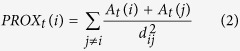


where *t* = the 1500 s or 2000 s and *d*_*ij*_ is the geographic distance between the two cells *i* and *j*.

The forest concentration index measures the total available forest in a focal grid cell and its four nearest neighbors (von Neumann neighbors) and is given by ref. 31


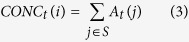


where *S* denotes a set containing the grid cell indices for the focal cell *i* and its four nearest neighbors and *t* = the 1500 s or 2000 s.

Like the connectivity index, both the proximity and concentration indices have been used to evaluate the degree of fragmentation of the local forests. A high proximity or concentration value suggests that the forest patches tend to be closely distributed across adjacent areas, while a low value suggests that the forest patches tend to be located in spatially disjunctive areas that are not adjacent to one another, implying a high level of forest fragmentation. In the figures and tables, forest connectivity is coded as IFM1500 and IFM2000, proximity as Prox1500 and Prox2000, and concentration as Conc1500 and Conc2000 for the past and current times, respectively.

### Spatial correlation test

Instead of applying a simple correlation test to detect evidence of extinction debt, a spatial correlation test with an adjustment of the degrees of freedom^34^,^35^ was implemented. This spatial correlation test can remove the artifacts caused by spatial autocorrelation and is thus very appropriate for our study at broad spatial scales. The R^36^ package “SpatialPack” (http://spatialpack.mat.utfsm.cl) was utilized to test the significance of spatial correlations between global vertebrate richness and forest variables.

### Semi-part correlation test

Because the formation of contemporary forest cover patterns may be partially attributed to past forest change, we also utilized a semi-part correlation test to control for the influences of other forest variables (e.g., forest connectivity) when investigating the correlations between global vertebrate richness patterns and a focal forest variable (e.g., Areas2000). Thus, the aim of the semi-part correlation test is to remove the influences of other forest cover-relevant variables on the focal variable.

### Partial correlation test

Because global species richness patterns might also be simultaneously affected by different forest cover-relevant variables, a partial correlation test was used to remove the joint influences of other forest cover-relevant variables on both the focal forest variable and the corresponding vertebrate richness.

The technical difference between semi-part and partial correlation tests rests with the targeted variables being controlled. Both species richness and the studied forest variable are the targets in the partial correlation test, while only the forest variable is the target in the semi-part correlation test. In other words, the influence of other variables on species richness will not be controlled for in a semi-part correlation.

The purpose of applying all three above correlation tests was to provide comparable and complementary evidence on the extinction debts for global forest-dwelling vertebrate species. Because multiple comparisons were involved in each of these correlation tests, the type I error may increase. To avoid this problem, a Bonferroni correction was also applied to ensure the significance of each correlation.

### Variable importance analysis

We conducted linear models with bootstrapping (10,000 iterations) to evaluate the relative importance of each forest cover-relevant predictor in explaining global terrestrial vertebrate richness patterns at the current time. We utilized the following metrics^37^, including the R^2^ contribution averaged over orderings among regressors (lmg), the contribution of each variable when included last in the model (last), the contribution of each variable when included first in the model (first), and the R^2^ decomposition according to a previous study^38^ (genizi). Please note that lmg, last, first and genizi here are the specific ranking methods for identifying the relative importance of the variables. The computing function for applying these metrics is “boot.relimp” in the R package “relaimpo” (https://cran.r-project.org/package=relaimpo).

### Estimation of spatial extinction debts

Assuming a stationary equilibrium is reached, the power-form SAR for global richness patterns across the three taxonomic groups in the 1500 s in grid cell *i* is given by


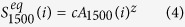


If we assume that the slope of the SAR model *z* does not change over time, then the species richness in the 2000 s can be calculated as


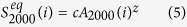


Therefore, we can infer that the species richness at equilibrium in the 2000 s is


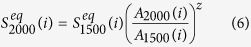


Therefore, the corresponding extinction debts would be the difference between the observed and expected species richness at equilibrium as follows:


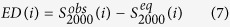


In the present study, 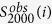 is the number of extant species found in cell *i* but not listed in the IUCN categories “EX” or “EW”. Negative extinction debts could occur based on the above equation (7), which may be interpreted as species or immigration credits^39^,^40^ but will be not discussed in detail in our study.

It should be noted here that 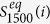 is the number of species found in cell *i* in the 1500 s, and its value is assumed to be very close to the total species found in cell *i* in the 2000 s. That is, 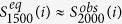.

It is challenging to define a proper *z* value when estimating species extinction across three taxonomic groups. One option is to choose empirical *z* values based on previous literature. The most common practice is to assume *z* = *0.25*^4^,^21^. Moreover, because SARs may over-estimate species extinction^41^, we also used other smaller z values (0.1 and 0.15) for comparison. Finally, we also fitted a *z* value for the SAR model of each taxonomic group, which is the slope of the log-transformed form of equation (4) as follows:


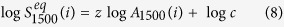


The estimated *z* value could be substituted back into equation (6) to calculate the expected species richness at equilibrium for the current period. Correspondingly, the number of species that are committed to extinction debts can be estimated by using equation (7).

### A comparison with IUCN-based extinction risk maps

The estimated extinction-debt patterns were compared to the summed extinction risk maps based on the IUCN Red List threatened categories. For the recently released IUCN Red List (2014 version), we followed previous studies^42^,^43^ with some modifications to assign a numeric probability value to each IUCN threatened category to indicate species’ extinction probabilities as the following: Data deficit (0.0001), Least concern (0.001), Near threatened (0.01), Vulnerable (0.1), Endangered (0.667), and Critically endangered (0.999). For each cell, we then computed the extinction risk as the sum of the extinction probabilities over all species that are found to occur in the cell. Such extinction risk maps are used to compare to the spatial extinction-debt patterns estimated based on SAR models.

## Additional Information

**How to cite this article**: Chen, Y. and Peng, S. Evidence and mapping of extinction debts for global forest-dwelling reptiles, amphibians and mammals. *Sci. Rep.* **7**, 44305; doi: 10.1038/srep44305 (2017).

**Publisher's note:** Springer Nature remains neutral with regard to jurisdictional claims in published maps and institutional affiliations.

## Supplementary Material

Supplementary Information

## References

[b1] Tilman D., May R., Lehman C. & Nowak M. Habitat destruction and the extinction debt. Nature 371, 65–66 (1994).

[b2] Lande R. Extinction thresholds in demographic models of territorial populations. Am. Nat. 130, 624–635 (1987).

[b3] Halley J., Sgardeli V. & Triantis K. Extinction debt and the species-area relationship: a neutral perspective. Glob. Ecol. Biogeogr. 23, 113–123 (2014).

[b4] Wearn O., Reuman D. & Ewers R. Extinction debt and windows of conservation opportunity in the Brazilian Amazon. Science (80-.). 337, 228–232 (2012). 10.1126/science.1219013 22798612

[b5] Helm A., Hanski I. & Partel M. Slow response of plant species richness to habitat loss and fragmentation. Ecol. Lett. 9, 72–77 (2006). 16958870 10.1111/j.1461-0248.2005.00841.x

[b6] Piqueray J. . Plant species extinction debt in a temperate biodiversity hotspot: community species and functional traits approaches. Biol. Conserv. 144, 1619–1629 (2011).

[b7] Soga M. & Koike S. Mapping the potential extinciton debt of butterflies in a modern city: implications for conservation priorities in urban landscapes. Anim. Conserv. 16, 1–11 (2013).

[b8] Bommarco R., Lindborg R., Marini L. & Ockinger E. Extinction debt for plants and flowering-visiting insects in landscapes with contrasting land use history. Divers. Distrib. 20, 591–599 (2014).

[b9] Cowlishaw G. Predicting the pattern of decline of African primate diversity: an extinction debt from historical deforestation. Conserv. Biol. 13, 1183 (1999).

[b10] Triantis K. . Extinction debt on oceanic islands. Ecography. 33, 285–294 (2010).

[b11] Jetz W., Wilcove D. & Dobson A. Projected impacts of climate and land-use change on the global diversity of birds. PLoS Biol. 5, e157 (2007). 17550306 10.1371/journal.pbio.0050157 PMC1885834

[b12] Leroy B. . Forecasting climate and land use changes, and protected areas: the contrasting case of spinders. Divers. Distrib. 20, 686–697 (2014).

[b13] Hof C., Araujo M., Jetz W. & Rahbek C. Additive threats from pathogens, climate and land-use change for global amphibian diversity. Nature 480, 516–519 (2011). 22089134 10.1038/nature10650

[b14] Barbet-Massin M., Thuiller W. & Jiguet F. The fate of European breeding birds under climate, land-use and dispersal scenarios. Glob. Chang. Biol. 18, 881–890 (2012).

[b15] Buckley L. & Roughgarden J. Biodiversity conservation: effects of changes in climate and land use. Nature 430, 1 (2004). 10.1038/nature02717 15233130

[b16] Bennett A. & Saunders D. In Conserv. Biol. All(Sodhi N. & Ehrlich P.) 88–106 (Oxford University Press, Oxford, 2010).

[b17] Hanski I., Zurita G., Bellocq M. & Rybicki J. Species-fragmented area relationship. PNAS, doi: 10.1073/pnas.1311491110 (2014). PMC3732936 23858440

[b18] Storch D., Keil P. & Jetz W. Universal species- and endemic-area relationships at continental scales. Nature 488, 79–81 (2012). 10.1038/nature11226 22722856

[b19] Dullinger S. . Extinction debt of high-mountain plants under twenty-first-century climate change. Nat. Clim. Chang. 2, 619–622 (2012).

[b20] Olivier P., van Aarde R. & Lombard A. The use of habitat suitability models and species-area relationships to predict extinction debts in coastal forests, South Africa. Divers. Distrib. 19, 1353–1365 (2013).

[b21] Thomas C. . Extinction risk from climate change. Nature 427, 145–148 (2004). 14712274 10.1038/nature02121

[b22] Thuiller W. . Uncertainty in predictions of extinction risk. Nature 430, 34 (2004). 10.1038/nature02716 15237465

[b23] Harte J., Ostling A., Green J. & Kinzig A. Climate change and extinction risk. Nature 430, doi: 10.1038/nature02718 (2004). 15237466

[b24] Ovaskainen O. & Hanski I. Transient dynamics in metapopulation response to perturbation. Theor. Popul. Biol. 61, 285–295 (2002). 12027615 10.1006/tpbi.2002.1586

[b25] Hanski I., Foley P. & Hassell M. Random walks in metapopulation: how much density dependence is necessary for long-term persistence? J. Anim. Ecol. 65, 274–282 (1996).

[b26] Ovaskainen O. & Meerson B. Stochastic models of population extinction. Trends Ecol. Evol. 25, 643–652 (2010). 20810188 10.1016/j.tree.2010.07.009

[b27] Ovaskainen O. & Hanski I. Extinction threshold in metapopulation models. Ann. Zool. Fennici 40, 81–97 (2003).

[b28] Hurtt G. . Harmonization of land-use scenarios for the period 1500-2100: 600 years of global gridded annual land-use transitions, wood harvest, and resulting secondary lands. Clim. Change 109, 117–161 (2011).

[b29] Jain A., Meiyappan P., Song Y. & House J. CO_2_ emissions from land-use change affected more by nitrogen cycle, than by the choice of land-cover data. Glob. Chang. Biol. 19, 2893–2906 (2013). 23529747 10.1111/gcb.12207

[b30] Poulter B. . Plant functional type classification for earth system models: results from the European Space Agency’s land cover climate change initiative. Geosci. Model Dev. 8, 2315–2328 (2015).

[b31] Watling J., Nowakowski A., Donnelly M. & Orrock J. Meta-analysis reveals the importance of matrix compositionfor animals in fragmented habitat. Glob. Ecol. Biogeogr. 20, 209–217 (2011).

[b32] Moilanen A. & Nieminen M. Simple connectivity measures in spatial ecology. Ecology 83, 1131–1145 (2002).

[b33] Hanski I. A practical model of metapopulation dynamics. J. Anim. Ecol. 63, 151–162 (1994).

[b34] Dutilleul P. Modifying the t test for assessing the correlation between two spatial processes. Biometrics 49, 305–314 (1993).

[b35] Clifford P., Richardson S. & Hemon D. Assessing the significance of the correlation between two spatial processes. Biometrics 45, 123–134 (1989). 2720048

[b36] R Development Core Team. R: A Language and Environment for Statistical Computing, Vienna, Austria. ISBN 3-900051-07-0, URL http://www.R-project.org. at http://www.r-project.org(2013).

[b37] Groemping U. Relative importance for linear regression in R: the package relaimpo. J. Stat. Softw. 17, 1–27 (2006).

[b38] Genizi A. Decomposition of R2 in multiple regression with correlated regressors. Stat. Sin. 3, 407–420 (1993).

[b39] Jackson S. & Sax D. Balancing biodiversity in a changing environment: extinction debt, immigration credit and species turnover. Trends Ecol. Evol. 25, 153–160 (2010). 19879014 10.1016/j.tree.2009.10.001

[b40] Hanski I. Extinction debt and species credit in boreal forests: modelling the consequences of different approaches to biodiversity conservation. Ann. Zool. Fennici 37, 271–280 (2000).

[b41] He F. & Hubbell S. Species–area relationships always overestimate extinction rates from habitat loss. Nature 473, 368–371 (2011). 21593870 10.1038/nature09985

[b42] Chen Y. Modeling extinction risk of endemic birds of mainland China. Int. J. Evol. Biol. 2013, 639635 (2013). 24455407 10.1155/2013/639635 PMC3878274

[b43] Gudde R., Joy J. & Mooers A. Imperiled phylogenetic endemism of Malagasy lemuriformes. Divers. Distrib. 19, 665–675 (2013).

