# Supplementary material for: Evidence and mapping of extinction debts for global forest-dwelling reptiles, amphibians and mammals
Source: Sci Rep. 2017 Mar 16;7:44305. doi: 10.1038/srep44305 (PMC5353668; doi:10.1038/srep44305)

**SUPPLEMENTARY INFORMATION**

**Evidence and mapping of extinction debts for global forest-dwelling reptiles, amphibians and mammals**

Youhua Chen, Shushi Peng

**Table S1.** Statistical significance of different correlation values between species richness and forest cover-related variables. Values outside the parentheses are the observed correlation coefficients computed empirically, while values inside the parentheses denote the 95% confidence interval of the distribution of correlation values calculated from 1000 randomized datasets. Observed coefficients in bold face and marked with asterisks represent that they are not significantly different from the random values.

|  | Reptiles | Mammals | Amphibians |
| --- | --- | --- | --- |
| Semi-part correlation coefficients and 95% confidence range | | | |
| Areas1500 | 0.105 (-0.02,0.021) | 0.055 (-0.022,0.02) | 0.12 (-0.024,0.019) |
| Areas2000 | 0.054 (-0.018,0.02) | **-0.015* (-0.019,0.018)** | **0.004* (-0.021,0.019)** |
| IFM1500 | 0.09 (-0.024,0.02) | 0.17 (-0.02,0.019) | 0.182 (-0.02,0.019) |
| IFM2000 | -0.029 (-0.022,0.022) | -0.055 (-0.022,0.02) | -0.031 (-0.026,0.019) |
| Prox1500 | -0.15 (-0.023,0.019) | -0.051 (-0.021,0.019) | -0.138 (-0.019,0.02) |
| Prox2000 | -0.07 (-0.02,0.019) | **0.01* (-0.021,0.02)** | **-0.01* (-0.023,0.019)** |
| Conc1500 | 0.03 (-0.021,0.022) | -0.035 (-0.019,0.018) | -0.027 (-0.02,0.02) |
| Conc2000 | 0.053 (-0.023,0.022) | 0.031 (-0.021,0.02) | 0.03 (-0.019,0.022) |
| Partial correlation coefficients and 95% confidence range | | | |
| Areas1500 | 0.109 (-0.02,0.021) | 0.07 (-0.022,0.02) | 0.142 (-0.024,0.019) |
| Areas2000 | 0.058 (-0.018,0.02) | **-0.019* (-0.019,0.018)** | **0.005* (-0.021,0.019)** |
| IFM1500 | 0.094 (-0.024,0.02) | 0.212 (-0.02,0.019) | 0.212 (-0.02,0.019) |
| IFM2000 | -0.031 (-0.022,0.022) | -0.072 (-0.022,0.02) | -0.037 (-0.026,0.019) |
| Prox1500 | -0.154 (-0.023,0.019) | -0.066 (-0.021,0.019) | -0.162 (-0.019,0.02) |
| Prox2000 | -0.075 (-0.02,0.019) | **0.013* (-0.021,0.02)** | **-0.012* (-0.023,0.019)** |
| Conc1500 | 0.031 (-0.021,0.022) | -0.044 (-0.019,0.018) | -0.032 (-0.02,0.02) |
| Conc2000 | 0.058 (-0.023,0.022) | 0.04 (-0.021,0.02) | 0.036 (-0.019,0.022) |
| Spatial correlation coefficients and 95% confidence range | | | |
| Areas1500 | 0.229 (-0.093,0.09) | 0.522 (-0.08,0.075) | 0.461 (-0.088,0.081) |
| Areas2000 | 0.16 (-0.083,0.09) | 0.46 (-0.081,0.078) | 0.421 (-0.083,0.087) |
| IFM1500 | 0.235 (-0.086,0.076) | 0.623 (-0.096,0.077) | 0.525 (-0.099,0.091) |
| IFM2000 | 0.159 (-0.087,0.086) | 0.546 (-0.079,0.079) | 0.473 (-0.084,0.085) |
| Prox1500 | 0.205 (-0.083,0.079) | 0.581 (-0.098,0.077) | 0.479 (-0.095,0.085) |
| Prox2000 | 0.137 (-0.082,0.085) | 0.519 (-0.08,0.078) | 0.442 (-0.083,0.085) |
| Conc1500 | 0.251 (-0.083,0.082) | 0.594 (-0.093,0.08) | 0.513 (-0.093,0.085) |
| Conc2000 | 0.174 (-0.086,0.085) | 0.518 (-0.081,0.082) | 0.462 (-0.083,0.08) |

**Fig. S1.** Current species richness patterns for global forest-dwelling reptiles (A), mammals (B) and amphibians (C). These maps are created using R package “raster” (version 3.2; https://www.r-project.org/).

A)


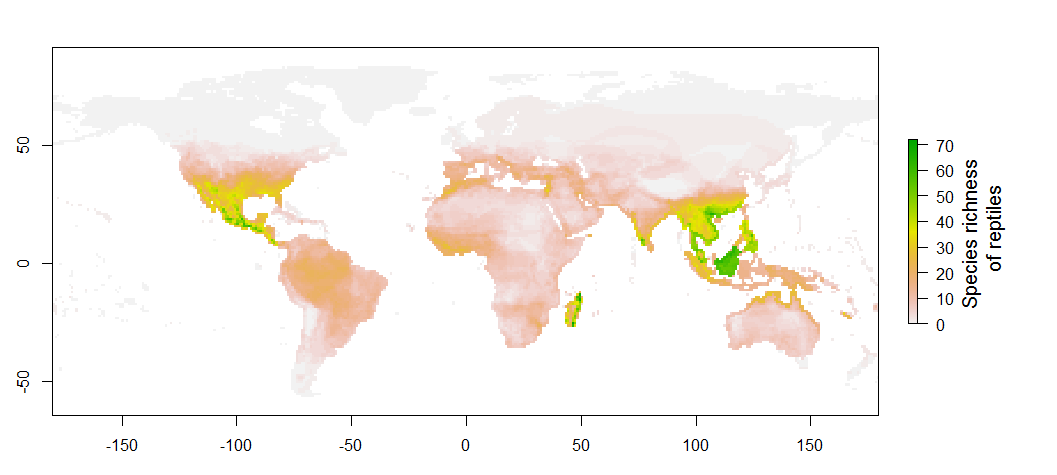


B)


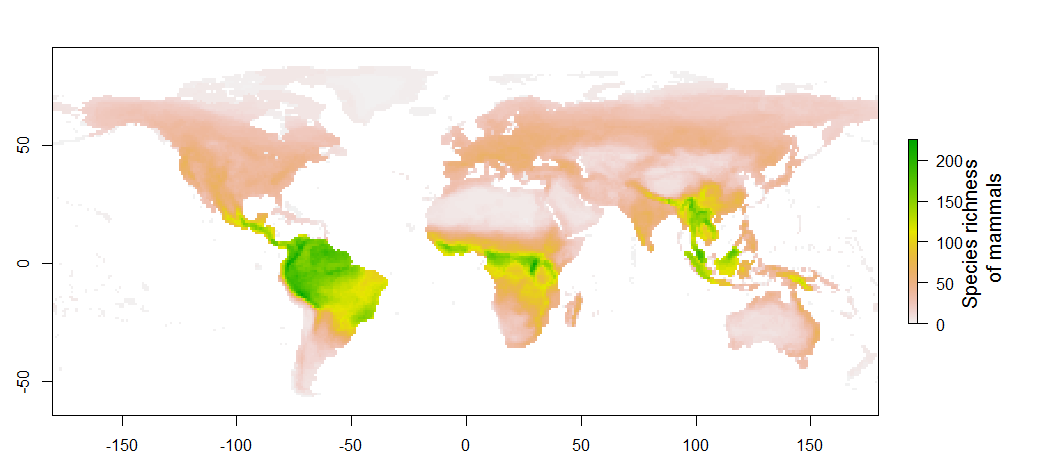


C)


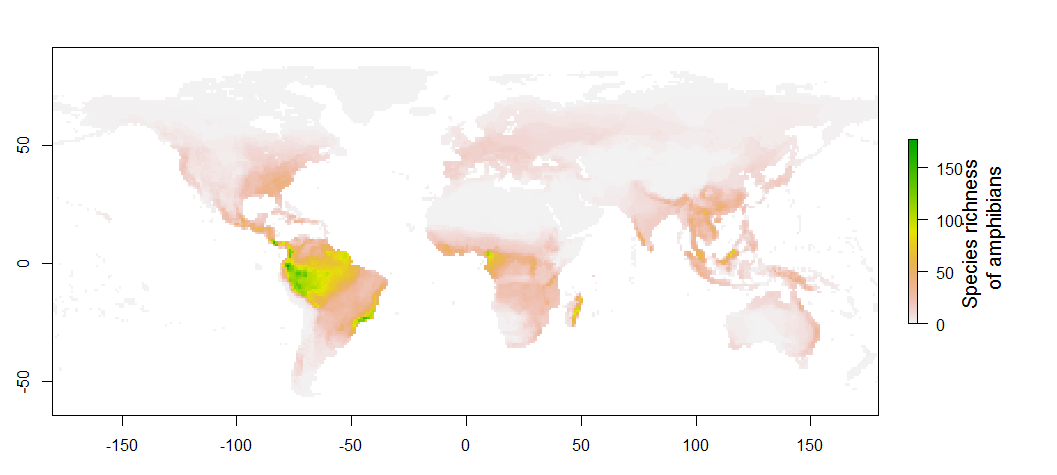


**Fig. S2.** Estimated spatial extinction debt magnitudes for global forest-dwelling reptiles (A-C), mammals (D-F) and amphibians (G-I) by using predefined small *z* values (*z*=0.1 and 0.15) or the fitted *z* value from the historical forest cover. In subplots A, D, and G, *z*=0.1; while in subplots B, E and H, *z*=0.15. At last, In subplots C, F and I, *z* values varied because they were directly fitted from the data. These maps are created using R package “raster” (version 3.2; https://www.r-project.org/).

A)


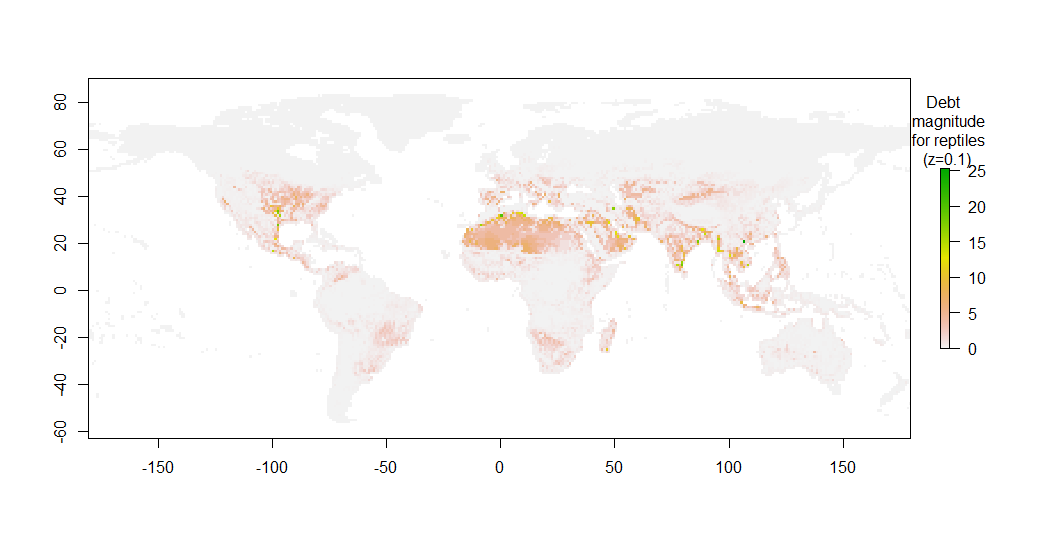


B)


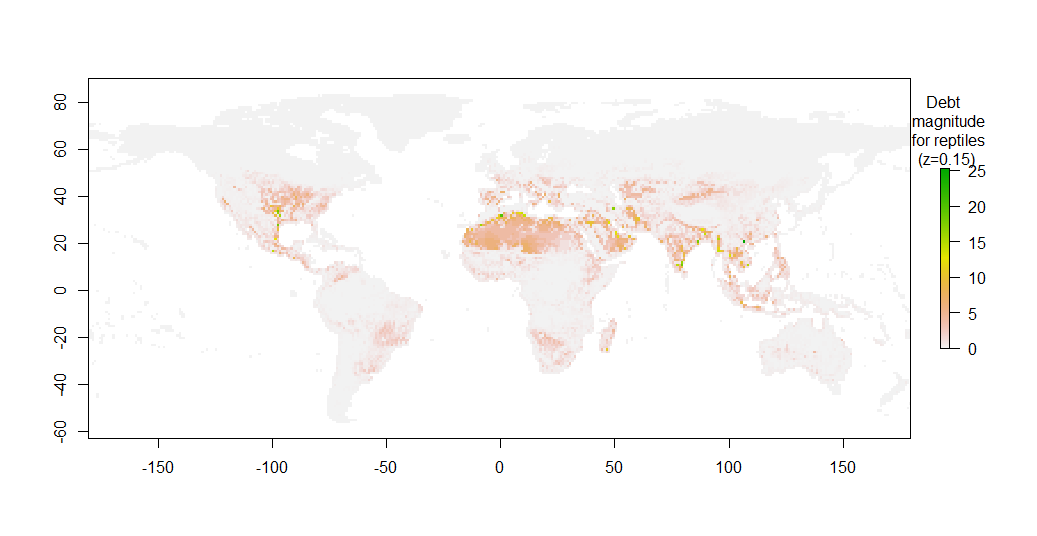


C)


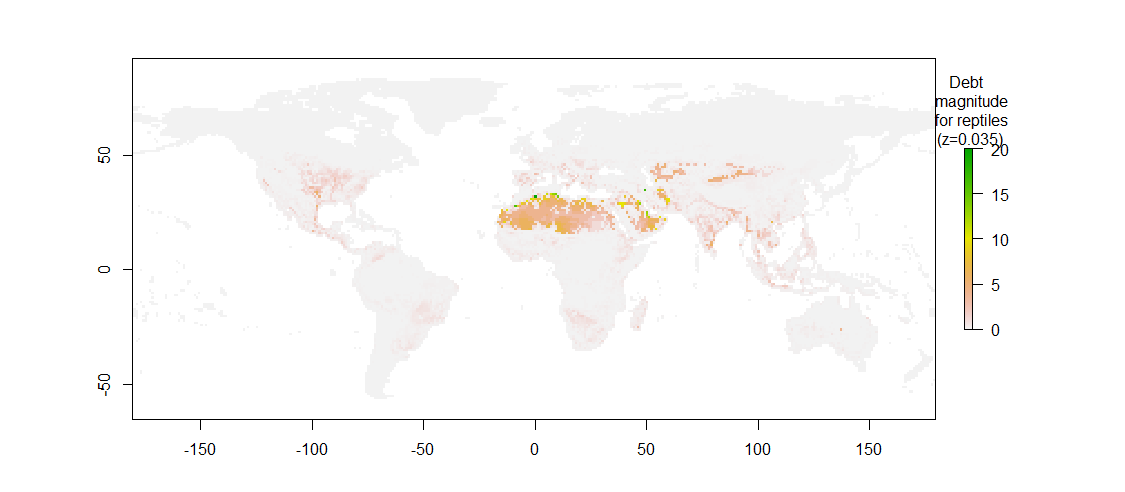


D)


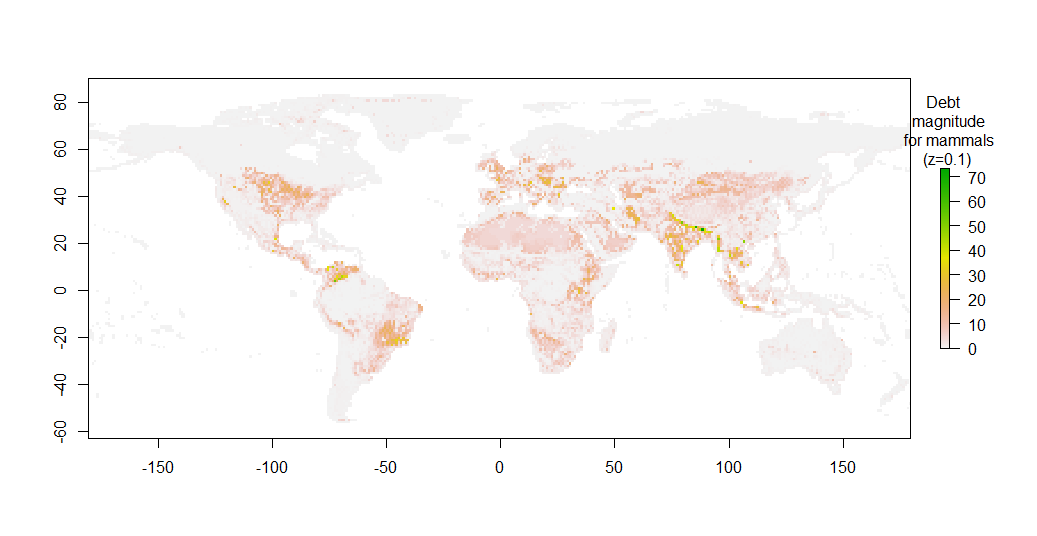


E)


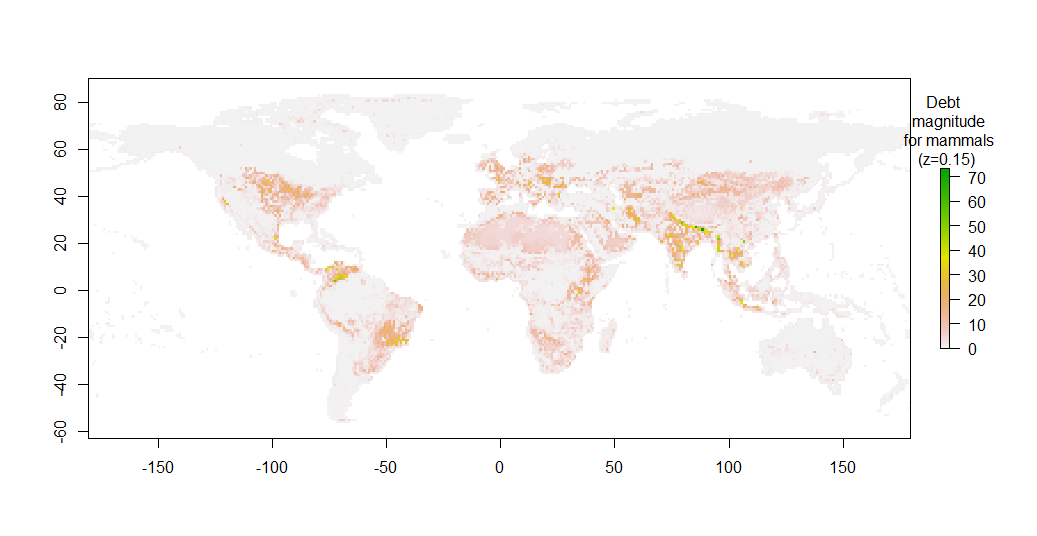


F)


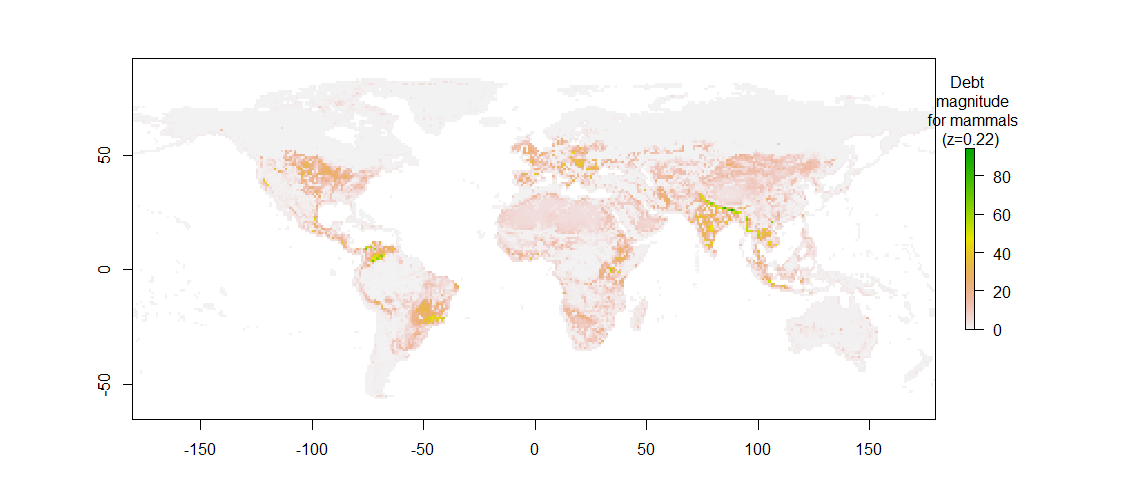


G)


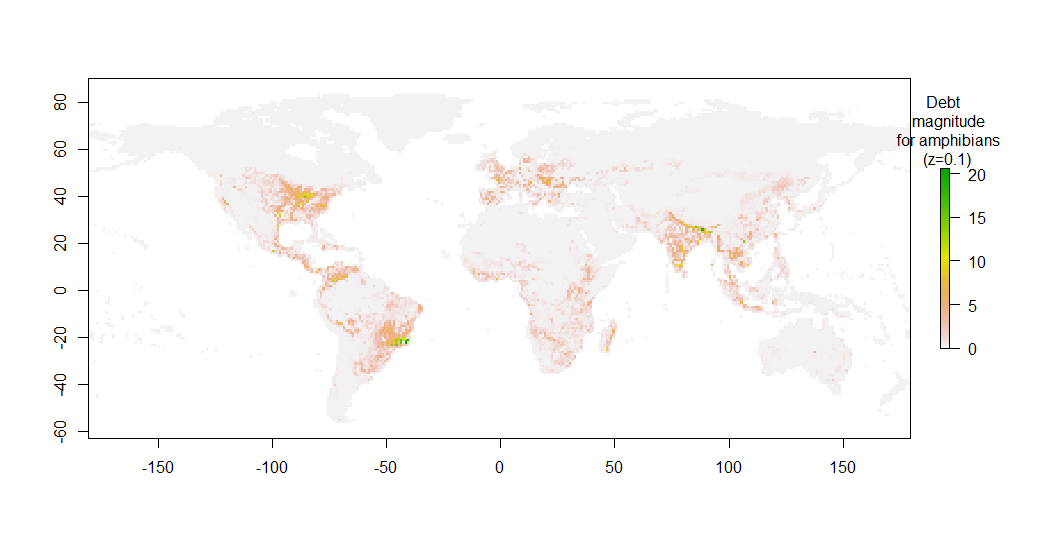


H)


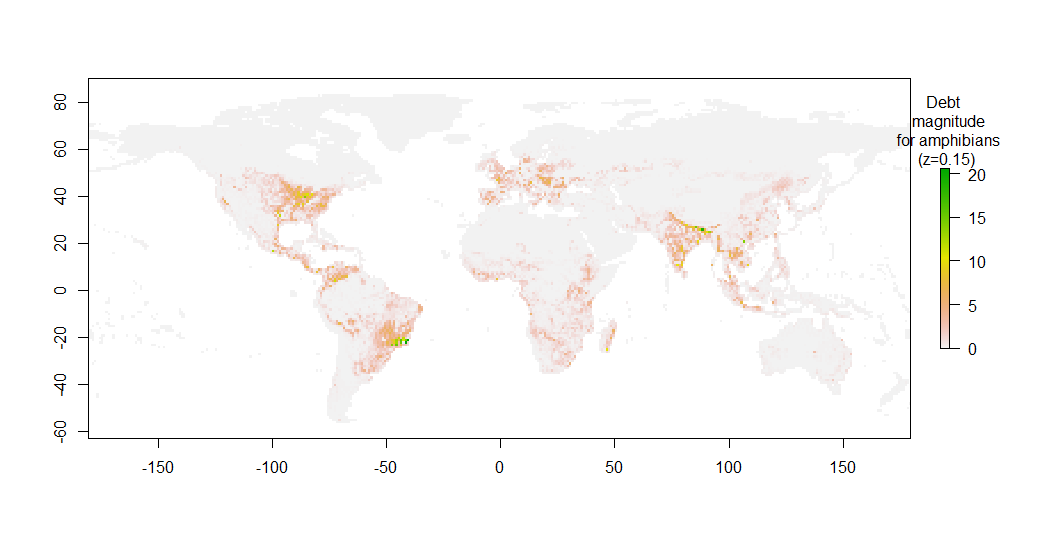


I)


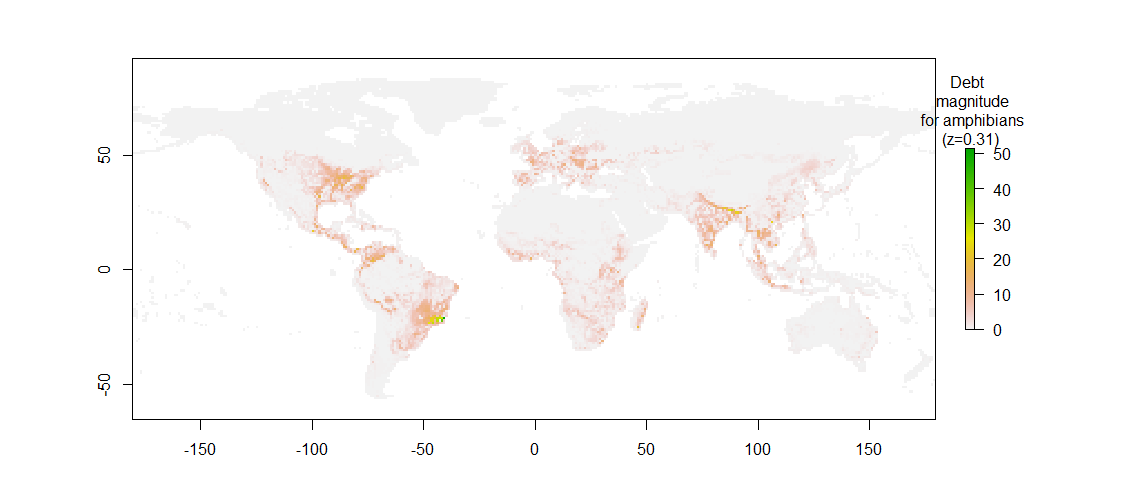


**Fig. S3.** IUCN-based extinction risk maps of global forest-dwelling reptiles (A), mammals (B) and amphibians (C). These maps are created using R package “raster” (version 3.2; https://www.r-project.org/).

A)


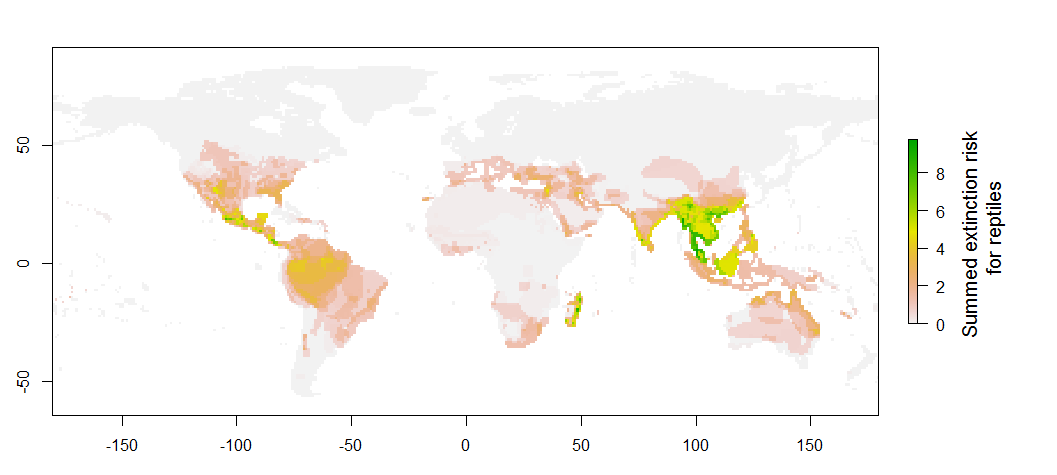


B)


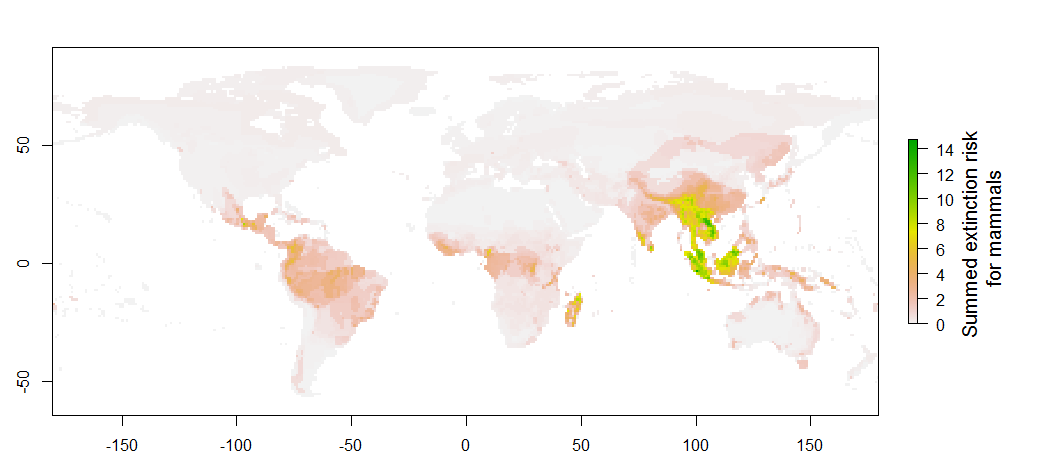


C)


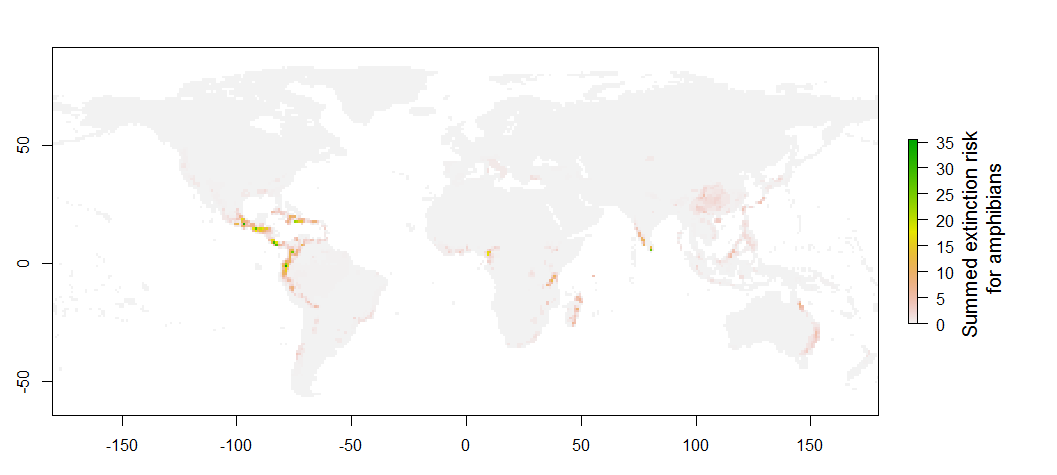

Supplement: Supplementary Information [file srep44305-s1.doc]
